# An ominous ECG

**DOI:** 10.1007/s12471-025-02008-4

**Published:** 2025-12-16

**Authors:** M. Libbrecht, T. De Meyer, M. Boulaksil

**Affiliations:** Department of Cardiology, Hartcentrum Kust, AZ Oostende hospital, Oostende, Belgium

## Answer

**Fig. 1:**
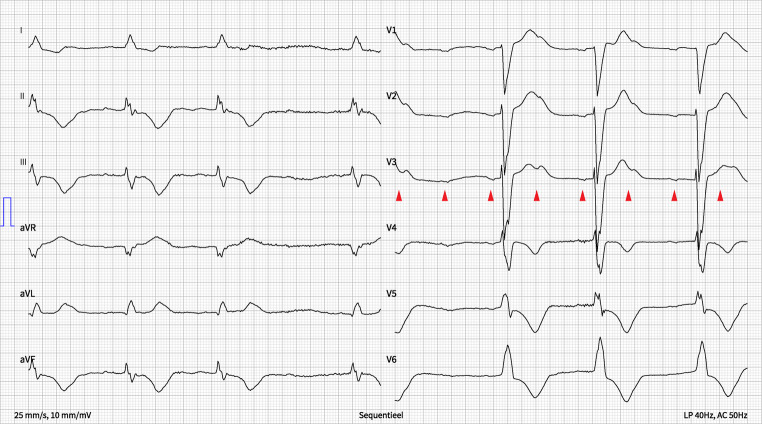
Initial ECG at presentation. Arrow heads indicate P waves, at exactly equal PP intervals

The ECG on admission (Fig. 1 in the Question) showed sinus rhythm, an intermediate electrical heart axis with a high-grade AV block, and a broad QRS complex bradycardia (QRS duration of approximately 185 ms with a ventricular rate of around 37 bpm). Because of the non-correlated P‑waves and the broad QRS complexes, we concluded a third-degree AV block with a ventricular escape rhythm (Fig. 1). There is a suggestion of group beating, based on which one could think of Wenckebach conduction. However, conduction behaviour here is very atypical for a Wenckebach type (multiple sequential non-conducted P waves [first 2 arrow heads], unequal PR intervals after blocked P waves [3rd vs 5th vs 7th arrow heads], and no shortening of the PR interval after a blocked P wave [7th arrowhead], all in Fig. 1).

Our differential diagnosis included intrinsic conduction system disease, drug-induced causes, acute myocardial ischaemia, and electrolyte disturbances. The blood potassium level was normal (4.4 mmol/L). Given the patient’s chronic use of flecainide, a sodium channel blocking (class Ic) antiarrhythmic drug, and the reduced kidney function, flecainide intoxication was initially suspected [1]. Blood was withdrawn at presentation for flecainide levels, and flecainide was discontinued.

**Fig. 2:**
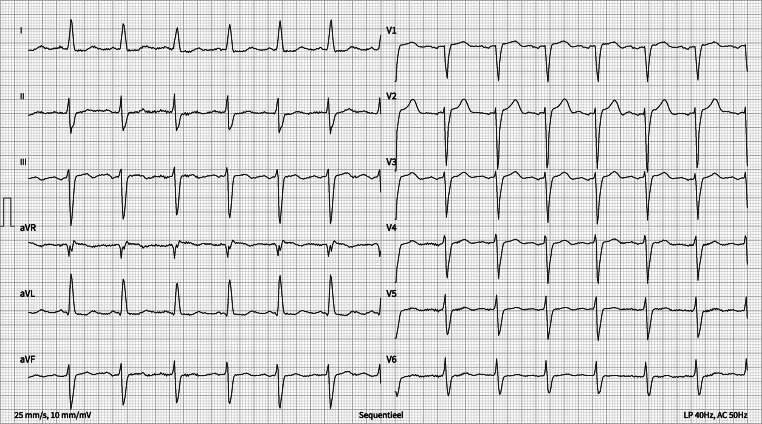
The ECG after revascularization

Because of the clinical presentation, the dynamic rise in troponin levels, and the patient’s history, we wanted to promptly exclude acute myocardial ischemia. Subsequent coronary angiography revealed significant in-stent restenosis in the venous bypass graft to the right coronary artery (See online supplement Fig. S1: video). Immediately after revascularization with drug-eluting balloons, the patient’s ECG improved (Fig. 2).

To our moderate surprise, flecainide plasma levels came back not elevated (360 ng/mL; normal therapeutic range for this lab 400–800 ng/mL). Therefore, because of the rapid ECG improvement after intervention, our initial diagnosis was reconsidered, and we became convinced that ischemia was the primary cause of the bradyarrhythmia since the right coronary artery supplies the atrioventricular node in most individuals [2].

This case emphasizes the diagnostic complexity in (elderly) cardiac patients on multiple drugs. Although drug toxicity must always be considered in acute conduction disturbances [3], it is crucial to maintain a broad differential diagnosis to guide appropriate management.

## Supplementary Information

**Online video** Coronarography on admission.
